# Herpes Simple Virus proctitis: a diagnostic dilemma between anal disorders

**DOI:** 10.11604/pamj.2019.33.33.17292

**Published:** 2019-05-16

**Authors:** Athanasios Syllaios, Spyridon Davakis

**Affiliations:** 1 First Department of Surgery, Laiko General Hospital, National and Kapodistrian University of Athens, Greece

**Keywords:** HSV, proctitis, anal

## Image in medicine

A 72-year-old man was referred to our surgical out-patient department because of severe anal pain, anal bleeding and itching for the last 5 days. Clinical examination revealed severe efflorescence with painful ulcers around the anus. Laboratory examinations revealed slightly elevated white blood cells count (WBC's= 13.000). There was taken liquid culture from the ulcer and sent for bacterial and virous analysis. The virus culture was positive for Herpes Simple Virus (HSV) type 2. The patient received valacyclovir 500mg per os for 10 days in order to reduce the risk for expansion of the disease as well as for minimizing the frequency and the duration of the relapses as there is no definite treatment. The patient was relieved from the symptoms and the rushing was diminished approximately ten days after the initiation of the appropriate medication. As a result, a high suspicion from physicians is required to diagnose HSV proctitis and surgeons should be aware of sexually transmitted anal diseases for the early detection and appropriate management of those patients, as patients may first appear to surgical out-patient departments seeking for help.

**Figure 1:**
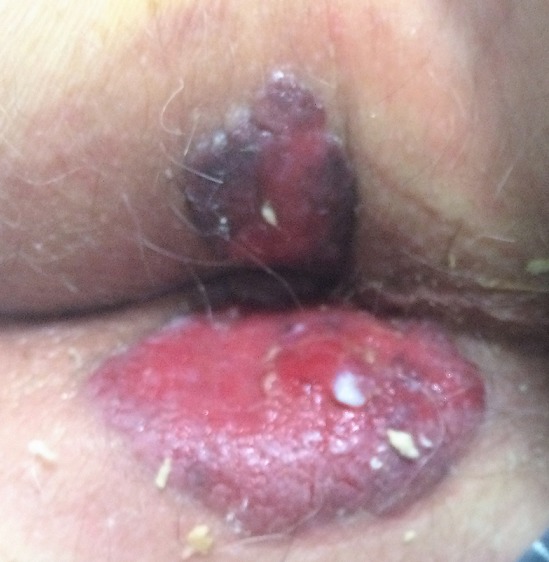
Herpes Simple Virus (HSV) proctitis on suspicious anal ulcer-lesion

